# Reference genes for quantitative Arabidopsis single molecule RNA fluorescence *in situ* hybridization

**DOI:** 10.1093/jxb/erac521

**Published:** 2022-12-29

**Authors:** Susan Duncan, Hans E Johansson, Yiliang Ding

**Affiliations:** John Innes Centre, Norwich Research Park, Norwich, UK; LGC Biosearch Technologies, 2199 S. McDowell Blvd, Petaluma, CA 94954, USA; John Innes Centre, Norwich Research Park, Norwich, UK; Instituto de Agrobiotecnología del Litoral, Argentina

**Keywords:** Arabidopsis root, housekeeping genes, mRNA quantification, smFISH, smRNA FISH, reference genes, standard genes

## Abstract

Subcellular mRNA quantities and spatial distributions are fundamental for driving gene regulatory programmes. Single molecule RNA fluorescence *in situ* hybridization (smFISH) uses fluorescent probes to label individual mRNA molecules, thereby facilitating both localization and quantitative studies. Validated reference mRNAs function as positive controls and are required for calibration. Here we present selection criteria for the first set of Arabidopsis smFISH reference genes. Following sequence and transcript data assessments, four mRNA probe sets were selected for imaging. Transcript counts per cell, correlations with cell size, and corrected fluorescence intensities were all calculated for comparison. In addition to validating reference probe sets, we present sample preparation steps that can retain green fluorescent protein fluorescence, thereby providing a method for simultaneous RNA and protein detection. In summary, our reference gene analyses, modified protocol, and simplified quantification method together provide a firm foundation for future quantitative single molecule RNA studies in Arabidopsis root apical meristem cells.

## Teaser

A modified protocol used to validate reference genes suitable for quantitative single molecule RNA fluorescence *in situ*hybridization studies in Arabidopsis root apical meristem cells.

## Introduction

Highly organized structural features of the Arabidopsis root apical meristem (RAM) make it a valuable model system for investigating how stem cells drive creation of differentiated tissues (Drapek *et al.*, 2017). The RAM consists of a stem cell niche located near the tip of the root that is protected from below by cells that are surrounded by a cap structure. A central vascular bundle extending shootward from the stem cells is surrounded by concentric cylinders of distinct cell types. Ongoing cell divisions in the RAM drive root growth, and differentiation is enforced as cells progress along opposing gradients of antagonistic phytohormones (Moubayidin *et al.*, 2013). In addition to an orderly axial arrangement, the RAM also consists of cell files that provide pseudo differentiation timelines. Over the last 20 years RAM mRNA studies have taken advantage of these robust architectural features to provide a fundamental understanding of how gene regulatory networks control stem cell maintenance, tissue patterning, and cell identity acquisition (Shahan *et al.*, 2021).

Standard reverse transcription–quantitative PCR (RT-qPCR) analysis can provide RAM mRNA abundance data, but as it generates relative averages for multiple cells, it is not suitable for investigating gene networks. To overcome this, microarray analysis and latterly RNA sequencing have been carried out on protoplasts sorted from lines with cell-type specific green fluorescent protein (GFP) expression (Birnbaum *et al.*, 2003; Brady *et al.*, 2007; Li *et al.*, 2016). However, issues regarding GFP expression specificity, cell sorting, and developmental variations caused unavoidable merging between some cell types and/or developmental states (Birnbaum, 2018). Single-cell RNA sequencing (scRNA-seq) has recently overcome these issues. This approach has greatly advanced our understanding of cell-type specific mRNA signatures and trajectories that underlie RAM development (Shahan *et al.*, 2022).

Although scRNA-seq has improved our fundamental understanding of how relative mRNA abundances across the transcriptome control root development, there are significant cellular resolution limitations to this technology. The term ‘single-cell sequencing’ suggests full transcriptome sequencing for every cell, but only a fraction of mRNA is captured and sequenced. This creates extensive cell-to-cell variability that must be taken into account by complex scRNA-seq normalization steps (Hafemeister and Satija, 2019). Outside of plant research, the superior specificity and sensitivity of single molecule RNA fluorescence *in situ* hybridization (smFISH) has made it a gold standard method for assessing trade-offs in scRNA-seq detection of rare cell expression variability (Torre *et al.*, 2018).

Direct mRNA visualization has revealed that many single and multicellular organisms have gene-specific mRNA abundances that broadly scale with cell size (Berry and Pelkmans, 2022). Maintenance of RNA concentration homeostasis requires crosstalk between transcriptional activity, cell size, and transcript abundance. There is evidence of this being achieved through kinetic coupling of mRNA synthesis, nuclear export, and transcript degradation rates. Quantitative single molecule mRNA imaging continues to be an important tool for investigating the mechanisms that underpin this complex aspect of gene regulation (Berry *et al.*, 2022; Berry and Pelkmans, 2022).

RNA localization is another aspect of gene regulation in which mRNA visualization has been important for investigating underlying mechanisms (Roden and Gladfelter, 2021). Many examples of subcellular spatial distribution patterns have been found to underpin processes in other systems, several of which may be pertinent to root development, e.g. cell polarity and asymmetric cell division (Das *et al.*, 2021). Although cell fractionation can reveal broad spatial differences, e.g. cytoplasmic versus nuclear and chromatin bound RNA (Jia *et al.*, 2020), imaging methods are key for high resolution spatial assessments in individual cells. Many fascinating insights have emerged from human, animal, and yeast fields relating to both mRNA transcript homeostasis and subcellular mRNA distributions. A slower uptake of single molecule RNA imaging is considered a key factor for the disproportionate number of examples reported for plants (Das *et al.*, 2021).

smFISH has been used extensively to determine subcellular mRNA localization and transcript abundance in a wide range of organisms. This method uses fluorescently labelled oligo probes to target and label RNA transcripts. Proximity of multiple probes hybridized to a single mRNA transcript generates a light diffraction limited spot of fluorescence that can be detected by standard widefield microscopy (Raj and Tyagi, 2010; Coassin *et al.*, 2014). Adaptation of this approach for RNA imaging in Arabidopsis RAM cells (Duncan *et al.*, 2016) has already provided unique insights into the regulation of a small number of plant genes (Ietswaart *et al.*, 2017; Rosa *et al.*, 2017; Sotta *et al.*, 2017; Questa *et al.*, 2020). In addition to supplying individual snapshots of cellular mRNA spatial distribution patterns, this method can generate high resolution images required for quantitative mRNA transcript homeostasis studies.

Reference genes are fundamental for robust quantitative gene expression analysis. This applies equally to both RT-qPCR (Bustin *et al.*, 2009) and image-based assessments (Deutsch *et al.*, 2008). Reference genes—also known as housekeeping, standard, or foundation genes—provide a control for variation in overall changes in gene expression beyond the gene of interest. For Arabidopsis, reference genes for RT-qPCR have been reported (Czechowski *et al.*, 2005; Dekkers *et al.*, 2012). Despite their importance for quantitative transcript imaging, smFISH reference genes have yet to be fully validated for plant experiments. Here we present design parameters and results from assessments of four reference gene candidates. As part of our validation process, we compared transcript abundance, mRNA concentrations, and relative fluorescence intensities. This revealed that probe sets designed to detect *TAP42 INTERACTING PROTEIN OF 41 KDA* (*TIP41*/AT4G34270), *MONENSIN SENSITIVITY1* (*MON1*/AT2G28390), and *ADAPTOR PROTEIN-2 MU-ADAPTIN* (*AP2M*/AT5G46630) mRNA are highly suited to quantitative calibration. In addition, we present a modified protocol and simplified quantification method that together provide a base for future Arabidopsis RAM single molecule mRNA studies.

## Materials and methods

### smFISH probe design

Each probe set was designed online to hybridize the coding sequence (CDS) using Stellaris Probe Designer, version 4.2 (LGC Biosearch Technologies, Petaluma, CA, USA). The following parameters were used: organism: other; filter level: 2; probe length: 20 nts; probe spacing: 2 nts. All probe sets were subjected to post design screening by batch blast of the Arabdopsis transcriptome at NCBI with settings for short input sequences, and as necessary, by alignment to multiple gene family transcripts. Individual probes with more than 3 out of 20 nucleotide mismatches were excluded from the final probe sets. T30 universal control probes (T30-Calflour 590-1 and T30-Quasar 570-1) were obtained from LGC Biosearch Technologies. Unlike the reference probe sets, 5ʹ and 3ʹ untranslated region (UTR) sequences were targeted together with the CDS to create maximum space for the odd and even sets of *MON1* mRNA probes. All probe sequences used are listed in Supplementary Table S1.

## Plant growth

Arabidopsis ecotype Col-0 seeds were surface-sterilized then sown in a row across the top half of a 10 cm square Petri plate on ½ MS–0.8% agar medium. After 2 d of stratification at 4 °C, the plates were then positioned upright in a PHCBI growth chamber (model MLR-352H-PE, PHC Corporation, Japan) set to 16-h light–8-h dark cycles at a constant 20 °C.

## smFISH protocol

The detailed protocol for Arabidopsis root meristem smFISH is available at Protocols.io (dx.doi.org/10.17504/protocols.io.rm7vzyworlx1/v1). Briefly, 5-day-old Col-0 seedlings were fixed in 16% methanol-free formaldehyde diluted to 4% in 1× phosphate-buffered saline (PBS; Thermo Fisher Scientific, Waltham, MA, USA) for 30 min then washed three times with 1× PBS. Root apical meristem squashes were created using manual pressure applied through a coverslip. After squashing, long forceps were used to grip the coverslip tightly onto the slide and the root sample was immersed directly into liquid nitrogen for around 10 s. Immediately after removal from the liquid nitrogen, the coverslip was removed carefully using a razor and the sample was left to defrost and dry for 1 h at room temperature. Slides were immersed in 70% ethanol for 1 h at room temperature. For a subset of samples, RNase treatment was carried out at this stage. Three 5 min 1× PBS washes were carried out on the slide before 12 U RNase A and 200 U RNase T1 (Thermo Fisher Scientific) diluted in 1× PBS was added to each slide. They were then incubated for 1.5 h at 37 °C followed by three 5-min washes with 1× PBS before the protocol was continued. Samples were equilibrated for 5 min with freshly prepared 10% (v/v) formamide Stellaris RNA FISH Wash Buffer A (LGC Biosearch Technologies). Probe stock of 250 nM was diluted 1:100 with freshly prepared 10% (v/v) formamide Stellaris Hybridization Buffer (LGC Biosearch Technologies) and incubated overnight at 37 °C in the dark. The buffer was removed in the morning and unbound probes removed by a 37 °C, 30-min incubation with Wash Buffer A. A second application of Wash Buffer A containing 1 µg ml^−1^ 4ʹ,6-diamidino-2-phenylindole (DAPI) was then applied and left at 37 °C for 30 min. After DAPI Wash Buffer A removal, Stellaris RNA FISH Wash Buffer B (LGC Biosearch Technologies), was added to each slide and left to equilibrate for 5 min at room temperature. After removal, ~20 µl Vectashield (Vector Laboratories, CA, USA) was added to the sample. A No.1 coverslip (VWR, Poole, UK) was then applied and sealed using CoverGrip (Biotium, CA, USA).

## Image acquisition and mRNA counts

A Zeiss Elyra PS1 inverted wide-field microscope fitted with an oil immersion ×100 (NA 1.46) lens and an Andor iXon 897 camera controlled by Zen 2.3 (Black) software was used to capture all image stacks. FIJI (Schindelin *et al.*, 2012) was used to quantify mRNA per cell using the following steps: maximum *z*-projection, manual outline of each cell region of interest (ROI; mean grey values and area measurements collected), subtraction of background with a rolling ball radius of 3 pixels, and finding of maxima command with a 1700 prominence setting. Approximate cell volumes were calculated as the product of the cell area, number of *z*-slices and 0.2 µm (*z*-section size). Bar charts and scatter plots were generated using Microsoft Excel (2210). Violin plots were created using the ggplot2 package (Wickham, 2016) in R. Pearson’s correlation coefficient analyses and one-way ANOVA–Tukey HSD post hoc tests were performed using the R functions cor.test(), aov(), and TukeyHSD(),respectively (RStudio Team, 2016).

## Probe set fluorescence assessment

It was first confirmed that each probe set detected a majority of mRNA in the cytoplasm, not the nucleus, as this is a requirement for quantitative calibration. Mean grey values for each probe set mRNA label were calculated from raw maximum *z*-projected stacks using FIJI (Schindelin *et al.*, 2012). Spots were selected from maximum *z*-projected images as 0.09 µm^2^ ROIs centred on the maximum value (i.e. three by three 100 nm pixel regions to cover ~250 nm diameter of each light diffraction limited spot). Twenty mean grey values were also calculated for background fluorescence generated by each probe set within 0.09 µm^2^ intracellular ROIs without mRNA labelling. Probe set comparisons were then made using total corrected cellular fluorescence (TCCF) calculations (McCloy *et al.*, 2014): integrated density−(area of selected cell×mean fluorescence). We calculated TCCF for each spot separately. To compare no-probe control and *MON1* mRNA samples, with and without RNase treatment, integrated densities were determined for identical ROI areas (59.94 µm^2^) centred on 12 individual nuclei for each experimental group.

## Results

### Reference gene selection and probe design

Extensive research regarding selection and validation of reference genes has provided a firm foundation for Arabidopsis RT-qPCR experiments (Czechowski *et al.*, 2005; Bustin *et al.*, 2009; Dekkers *et al.*, 2012). We applied similar principles to identify a panel of robust smFISH reference probe sets. Our initial panel of candidate genes were selected from a list of previously published qPCR reference standards identified from Affymetrix ATH1 whole-genome GeneChip studies (Czechowski *et al.*, 2005; Dekkers *et al.*, 2012). This list contained hundreds of Arabidopsis genes with superior expression stability throughout development and under a range of environmental conditions.

Potential candidates were then screened using more up-to-date genomic and transcriptomic data. Firstly, near ubiquitous expression across different tissues and stages of development was checked using the TRAVA RNA-seq database (Klepikova *et al.*, 2016). Next, we checked the Araport11 genome assembly (Cheng *et al.*, 2017) to identify candidates from our list that: (i) are more than 700 nucleotides long, excluding the polyA tail, (ii) exist as a single copy in the genome or are evolutionarily distinct from other paralogues and pseudogenes, (iii) have minimal transcript variants, i.e. genes with alternative transcription start sites, alternative splicing, and alternative polyadenylation sites, and (iv) contain minimal regions of common exonic sequence that can be targeted by an inclusive probe set to minimize off-target binding potential.

Notably, several of the classic reference genes, *GAPDH*, *ACTIN* (*2* and *8*), *UBQ10* (*UBIQUITIN*), and *TUBULIN* (*4* and *6*) failed our selection criteria for Arabidopsis smFISH. In addition, the gene abbreviated to *PP2A* in previous Arabidopsis smFISH publications (*PHOSPHOPROTEIN PHOSPHATASE REGULATORY SUBUNIT 2A PROTEIN*, AT1G13320*/*PP2AA3) was also excluded from our candidate list due to its close homology to additional *PP2A* subunits, *PP2AA2* (AT3G25800) and *RCN1* (AT1G25490).

The top candidates we selected for probe design were: AT2G28390 (*MON1*), AT4G34270 (*TIP41*), and AT5G46630 (*AP2M*). We also included AT4G35800, the largest subunit of nuclear DNA-dependent RNA polymerase II (*NRPB1*) as homologues of this gene are routinely used as human and mouse smFISH reference genes. Although the previously published Arabidopsis control gene AT1G13320 (*PP2A*) did not meet our now more stringent selection criteria, we included it here for comparison. The five probe sets contained between 43 and 48 oligos. All probe sequences are listed in Supplementary Table S1, and probe design details are included in ‘Materials and methods’.

### smFISH protocol modifications

Modifications to the previously published Arabidopsis protocol (Duncan *et al.*, 2016) were prompted by delayed supply of mounting-medium enzymes. Here we outline protocol amendments that make it compatible with the convenient commercial antifade mountant Vectashield. In addition, we used a simple PBS 4-fold dilution of pre-mixed 16% methanol-free formaldehyde to produce fixed cells indistinguishable from those fixed by freshly prepared paraformaldehyde. This not only reduces the overall protocol time but also removes many of the safety risks associated with fresh paraformaldehyde preparation. Use of commercially available nuclease-free wash and hybridization buffers were found to further reduce reagent preparation time without compromising mRNA image quality (see Protocols.io, dx.doi.org/10.17504/protocols.io.rm7vzyworlx1/v1).

For effective detection, true probe signal intensities must surpass both inherent tissue autofluorescence and staining created by off-target and unbound probes. Varying the length of wash steps did not reduce overall background fluorescence suggesting that our optimized protocol efficiently removes unbound probes from the sample. Empirical observations from our experimental set-up suggest that prolonging any of the wash steps or reducing either the 5-min formamide equilibration before probe hybridization or the 5-min formamide removal wash before addition of Vectashield leads to a reduction in mRNA label intensity.

### mRNA detection and quantification

Despite a lack of chlorophyll, fixed plant roots still emit a level of autofluorescence that makes high resolution imaging a challenge, especially for low level fluorescent labels (Dumur *et al.*, 2019). To overcome this for smFISH, a root squash method can be used to create an array of detached RAM cells that are suitable for two-dimensional mRNA quantification (Duncan *et al.*, 2017) (Supplementary Fig. S1). Although a squash typically creates regions of both single- and multi-cell layers (Supplementary Fig. S1B), without optical tissue clearance, the quality of individual mRNA labelling in overlying cells is typically poor (Zhao *et al.*, 2022, Preprint). For our reference validation study, we have focussed on single layer regions and used straightforward quantitative analysis steps (Supplementary Fig. S1C).

Five-day-old Col-0 seedling roots grown under standard conditions were used to evaluate our probe sets (see ‘Materials and methods’ for details). Since we have observed some artefacts in older seedlings, we recommend stringent control experiments are conducted for quantitative RNA assessments of older plants. Morphological features indicate that single-cell-layer regions mostly contain epidermis, cortex, and endodermis cells. Stele, pericycle, cortex/endodermis initials, and quiescent centre cells are also present at lower frequencies (Supplementary Fig. S1A). Although root cap cells are also often abundant in single-cell-layer regions, they were not included in this study due to poor probe penetration. This is presumably due to enhanced cell wall integrity required for robust protection of the root tip (Kumpf and Nowack, 2015). Furthermore, cells located further up the root in the transition and elongation regions were also excluded due to a high frequency of non-specific labelling (Duncan *et al.*, 2016). For this study, only cells containing a clear nucleolus and located in single-layer regions were assessed. These rules effectively ruled out assessments of root cap cells and cells located outside of the RAM region.

Although it is straightforward to visualize high intensity spots of fluorescence in raw smFISH images, probe binding efficiency is not identical for every transcript. Partial transcript degradation, RNA structure, binding proteins, and ribosome masking can create variation in mRNA fluorescence intensities. Ultimately at a certain point, low level mRNA signals can no longer be distinguished from background fluorescence. Therefore, effective image thresholding must be applied to maximize true signal counts, whilst minimizing false positive detection rates.

The automated smFISH image analysis pipeline FISHcount has proven invaluable to previous quantitative mRNA experiments for Arabidopsis (Duncan *et al.*, 2016, 2017). However, installation problems have been reported and the underlying processes used for spot detection may not be intuitive to researchers new to microscopy. To alleviate initial concerns regarding label ambiguity, we devised a simple three step process using FIJI (Schindelin *et al.*, 2012) that removes background fluorescence to leave mRNA signals suitable for automated quantification (for step-by-step instructions, see Protocols.io, dx.doi.org/10.17504/protocols.io.rm7vzyworlx1/v1). This straightforward processing method was used to generate quantitative results for our reference probe sets.

**Fig. 1.**
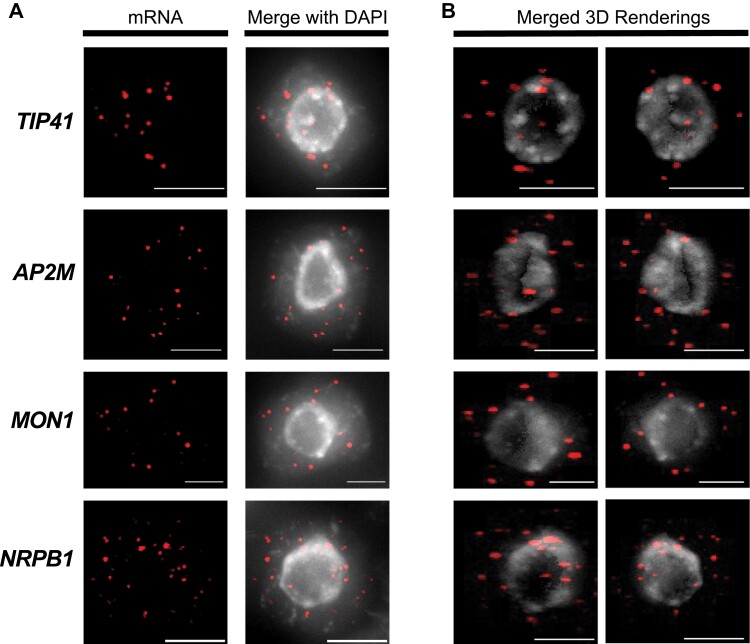
Representative mRNA spatial distributions of four reference gene candidates. (A) Representative maximum intensity *z*-projected images for each reference gene. (B) *x*/*z* orthogonal views through the same root apical meristem cells following FIJI 3D volume rendering. All images were processed using background subtraction (see ‘Material and methods’ for details). Brightness and contrast adjustments were made to improve figure clarity only. Red, fluorescent mRNA labels; grey, nuclear stain DAPI. Scale bars: 5 µm.

We observed that all four of our candidate probe sets generated clear spots of fluorescence that were enhanced by background subtraction. Example maximum *z*-projected images for all four probe sets showing representative labelling patterns are given in Fig. 1A. In addition to quantitative mRNA data, examples of *x*/*z* orthogonal views from 3D volume rendered images also demonstrate the potential of this method for generating spatial mRNA distribution data (Fig. 1B).

We also provide example images for T30 positive control probe sets that can be useful when setting up smFISH for the first time (Supplementary Fig. S2). They consist of 30 consecutive thymine nucleotides that bind polyA tails to generate diffuse labelling wherever mRNA is present. An expected the T30 labelling pattern has higher florescence in the nucleus compared with the cytoplasm and very low staining in the nucleolus. Several dye options are available for these probes so they can be useful for testing different microscope set ups. T30 probes and not quantitative, but they can be multiplexed with other probe sets to generate cell outlines suitable for automated cell segmentation.

### Reference mRNA counts per cell

**Fig. 2.**
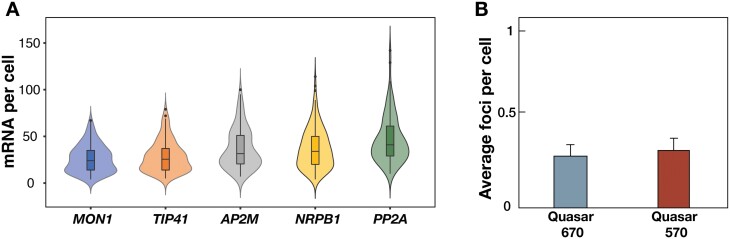
Variation in reference gene mRNA counts per cell. (A) mRNA counts per cell were determined for each reference gene using complimentary smFISH probes. Violin plots show overall data distribution. Box plot sections indicate the median, 25th, and 75th percentiles, and whiskers indicate minimum and maximum values. (B) Bar charts showing means from control experiments with smFISH probe omission. Microscope settings used to detect two common smFISH dyes revealed average false detection rates of one mRNA per approximately three to four cells. *n*≥93 in (A) and *n*=68 in (B). Error bars: +SEM.

An important smFISH reference consideration is transcript abundance. Probe sets that label less than 10 transcripts per cell typically have higher relative background fluorescence levels that can make detection more challenging. Although higher transcript abundances are generally associated with higher relative label intensities, more than 100 mRNAs per cell can cause optical crowding and hamper mRNA quantification, particularly from 2D images. When we analysed our four candidate reference genes, alongside the previously published control *PP2A*, our results confirmed that all sets met transcript abundance requirements, i.e. greater than 10, but lower than 100. The median number of mRNAs detected per cell was: 26, 29, 37, 37, and 46 for *MON1*, *TIP41*, *AP2M*, *NRPB1*, and *PP2A*, respectively (Fig. 2A).

Examination of no-probe control samples revealed only a single non-specific signal approximately every three cells for both far-red Quasar 670 and Quasar 570 dye channels (Fig. 2B; Supplementary Fig. S3A). We also observed that RNase treatment depleted T30 labelling and removed *MON1* mRNA signals (Supplementary Fig. S3B, C). A further control experiment was carried out with alternating probe dyes to test specificity. For a fair test, two sets of 48 probes would have bound to the coding sequence of each transcript. However, even when we expanded the target space to include 5ʹ and 3ʹ UTRs of *MON1* mRNA, only 34 probes could be assigned to each ‘odd’ and ‘even’ set (Supplementary Fig. S4A). This resulted in a 25% reduction in intensity compared with *MON1* mRNA reference spots. The potential for probe masking was also increased due to a higher probability of RNA binding proteins located in UTRs (Gebauer *et al.*, 2012). Despite these challenges, we detected 77% mean co-localization for alternating probe dyes for each *MON1* mRNA label (Supplementary Fig. S4B, C).

### Reference mRNA concentrations

A constant level of mRNA cellular concentration reflects highly regulated gene homeostasis (Kempe *et al.*, 2015; Padovan-Merhar *et al.*, 2015; Ietswaart *et al.*, 2017; Berry and Pelkmans, 2022). An ideal reference gene would therefore have highly regulated homeostasis that ensures minimal variations in mRNA concentration. The root apical meristem consists of a range of cell types, each with a different morphology (Supplementary Fig. S1). Although we predict our reference standard gene candidates to exhibit low variation across the range of cell types imaged, differences in cell size make mRNA counts per cell less informative than cellular concentrations.

We collected mRNA counts per cell for each probe set group that contained cells spanning a comparable size range (Supplementary Fig. S5). Cells in the *MON1*, *TIP41*, *AP2M*, *NRPB1*, *PP2A* and no-probe control groups had average cell volumes of 1191, 1233, 1278, 996, and 1204 µm^3^, with standard deviation measurements of 536, 506, 604, 495, 544, and 601 µm^3^, respectively. This ensured comparable mRNA concentration calculations could be completed for each probe set.

**Fig. 3.**
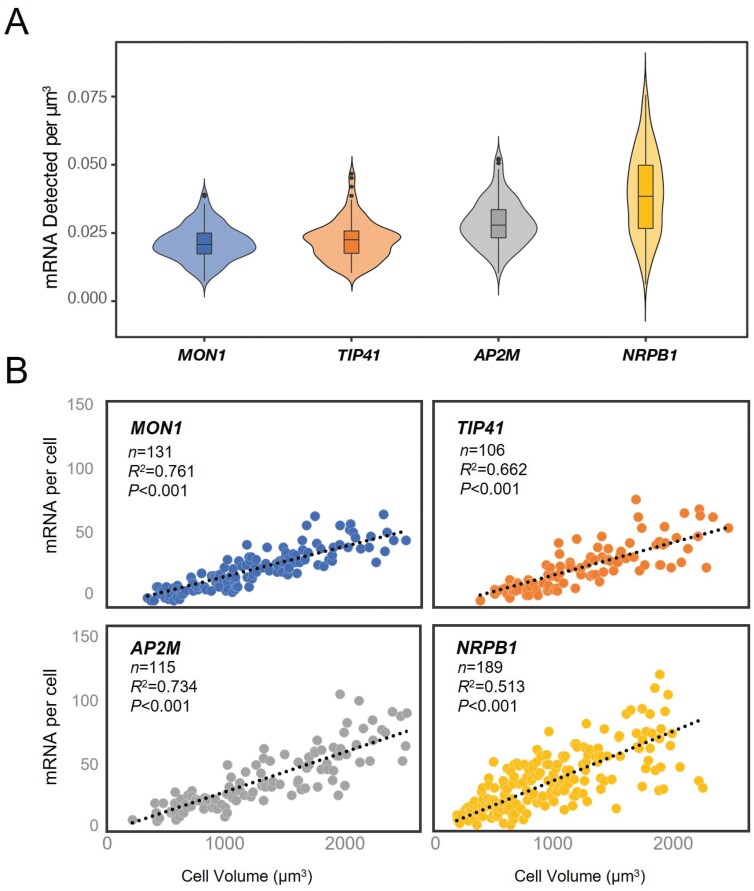
Reference gene mRNAs per cell correlate with cell size. (A) Violin plots showing distribution of mRNA counts per µm^3^ cell volume for each standard. Box plot sections indicate the median, 25th, and 75th percentiles, and whiskers indicate minimum and maximum values. (B) Scatter plots revealing variations in mRNA counts across a range of cell sizes. Pearson’s correlation coefficient statistics are presented for each probe set.

Mean cellular mRNA concentrations for *MON1* and *TIP41* were found to have the lowest mean concentrations (0.021 and 0.023 mRNA per µm^3^, respectively). *AP2M* was identified as the intermediary reference with a mean concentration of 0.288 mRNA per µm^3^. Higher average concentrations were calculated for *NRPB1* and *PP2A* (0.390 and 0.0391 mRNA per µm^3^, respectively (Fig. 3A; Supplementary Fig. S6A). Next, we explored concentration variation across a similar range of cell sizes by first generating scatter plots (Fig.3B; Supplementary Fig. S6B). Pearson’s correlation coefficient analyses for each set revealed that *MON1* mRNA had the highest correlation between mRNA and cell volume (*R*^2^=0.761, *P<*0.001, Fig. 3B). A lower correlation was calculated for *AP2M* (*R*^2^=0.734, *P<*0.001) and *PP2A* (*R*^2^=0.778, *P<*0.001). A broader but acceptable variation was calculated for *TIP41* (*R*^2^=0.662, *P<*0.001), and the lowest correlation was observed for *NRPB1* (*R*^2^=0.513, *P<*0.001).

### Reference mRNA label intensity

**Fig. 4.**
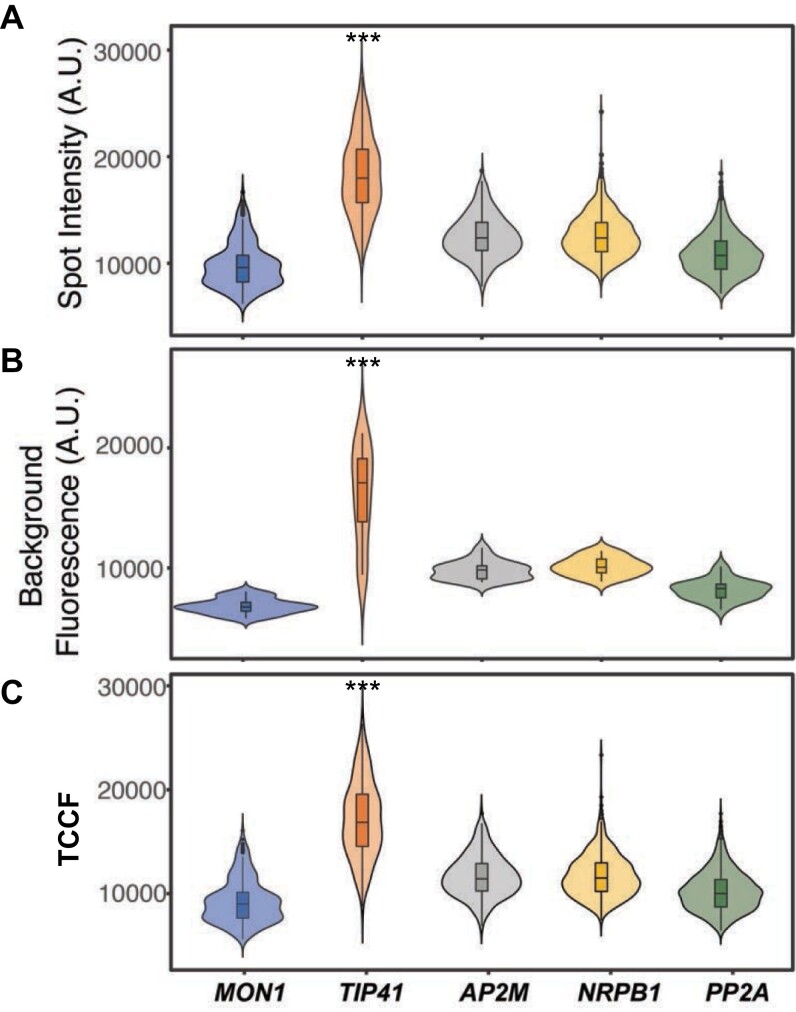
The *TIP41* probe set generates the highest relative spot intensity. Violin plots in sections (A–C) present averages and data distributions for reference mRNA spot intensities, background fluorescence, and total corrected cellular fluorescence (TCCF), respectively. Box plot sections indicate the median, 25th, and 75th percentiles, and whiskers indicate minimum and maximum values. For background fluorescence estimation: *n=*20; spot intensity and TCCF: *n*≥182. ***P<0.001, one-way ANOVA–Tukey HSD pot hoc test results.

Fluorescence intensity is an important consideration for a reference probe set. All sets had a maximum number of probes designed to tile along each transcript whilst avoiding problematic sequences that could either impede probe hybridization or increase off-target binding. All our reference candidate sets met stringent design criteria but had different emitted raw spot fluorescence intensities. *TIP41* mRNA was found to emit the highest average intensity. The lowest average was seen for *MON1*, with measurements for *AP2M*, *NRPB1*, and *PP2A* mRNA between these two extremes (Fig. 3A). However, clear smFISH labelling relies on the ability to distinguish true mRNA labels above background fluorescence, i.e. inherent autofluorescence plus additional signal created by unavoidable off-target binding and residual unbound probes. So next we assessed average background fluorescence levels for each set. In addition to mRNA emitting the highest intensity fluorescence, the *TIP41* probe set also generated the highest level of background. Consistent with the lowest mRNA signal intensity, the lowest background level was also seen for *MON1* probes, with intermediary levels observed for the other three sets. For overall comparisons, we calculated TCCF values to take account of both raw spot and background intensities (McCloy *et al.*, 2014). A one-way ANOVA–Tukey HSD post hoc test revealed that the *TIP41* mRNA spots generated the highest mean TCCF, whilst *MON1*, *AP2M*, *NRPB1*, and *PP2A* provided 40%, 38%, 34%, and 26% of *TIP41* relative intensity, respectively (Fig. 4).

### GFP fluorescence retention following smFISH protocol

**Fig. 5.**
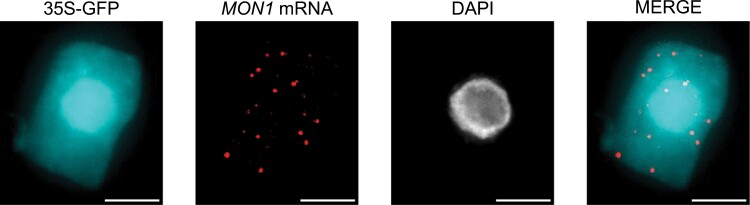
The Arabidopsis root smFISH protocol preserves GFP fluorescence. Maximum *z*-projection image of representative root apical meristem cell demonstrating *MON1* mRNA detection (red) together with constitutively expressed GFP (cyan) and the nuclear stain DAPI. Scale bars: 5 µm.

Single molecule mRNA and protein can be imaged in the same cells using sequential smFISH and immunocytochemistry protocols. However, we found that the mild temperature and chemical treatments required for our smFISH RNA detection protocol retains GFP signal in transgenic Arabidopsis lines with high levels of expression, e.g. GFP driven by a 35S promoter (Fig. 5). This opens the potential for expedited mRNA and protein detection in transgenic lines that emit sufficient GFP fluorescence intensity. Although GFP marker lines could be used to aid cell-type identification, developmental differences may make this approach unsuitable for direct comparisons (Birnbaum, 2018). Furthermore, studies assessing mutant effects would require additional lines to be generated with these markers. To overcome this, we recommend use of multiplexed probe sets to simultaneously label cell-type specific RNA. Machine learning algorithms applied to single cell transcriptome datasets have recently expanded the pool of suitable gene candidates (Yan *et al.*, 2022).

## Discussion

Single molecule detection of RNA using smFISH has been proven in many systems to be a robust quantitative method of determining transcript quantity and subcellular localization (Asp *et al.*, 2020; Das *et al.*, 2021). The aim of our study was to design and validate probe sets for use as Arabidopsis reference standards to support future smFISH studies in the RAM. We applied stringent design rules to genes with low levels of mRNA perturbation reported during development or in response to biotic and abiotic stress. After sequence-associated factors ruled out several candidates, we selected four gene probe sets for testing: *MON1*, *TIP41*, *AP2M*, and *NRPB1*. Our modified smFISH protocol and simplified image analysis method was then used to calculate transcript abundance, cellular concentration, and mRNA label intensities. This process revealed broad cytoplasmic transcript spatial distributions for all sets (Fig. 1). Furthermore, we confirmed that all candidates met our mRNA abundance criteria (10–100 transcripts per cell) making them all suitable for simple 2D quantification (Fig. 2A). It should be noted that quantification of mRNA with higher abundance can also be achieved by three-dimensional processing, e.g. the FIJI plugin RS-FISH (Bahry *et al.*, 2022).

Broad scaling of gene-specific mRNA abundance with cell size has been reported in many single and multicellular organisms (Berry and Pelkmans, 2022). The single smFISH investigation into this phenomenon in plants reported modulation of *FLC* mRNA homeostasis by transcription of the antisense long non-coding RNA *COOLAIR* (Ietswaart *et al.*, 2017). Given that this is a report for an important flowering time gene, it is likely that other modulations also exist in the RAM to specifically regulate root development. Data presented here confirm that *MON1*, *TIP41*, and *AP2M* probe sets would all be suitable for calibrating future studies investigating RAM mRNA homeostasis (Fig. 3B). However, larger variation in mRNA concentration observed in larger cells for all probe sets suggests that the most accurate data would be obtained for cells smaller than ~1500 μm^3^.

RNA localization is a pervasive mechanism controlling many aspects of gene regulation (Langdon and Gladfelter, 2018). smFISH has already revealed three examples of RNA localization in Arabidopsis RAM cells: long non-coding intronic *COOLAIR* RNA accumulations around *FLC* loci during vernalization (Rosa *et al.*, 2017), *NIP5;1* mRNA retention at sites of transcription (Sotta *et al.*, 2017), and *SHORT ROOT* RNA aggregations in a subset of RAM cells (Zhang *et al.*, 2019). Tian *et al.* (2020) have reported further examples for other plant tissues. However, given the prevalence of RNA localization mechanisms identified in other organisms, there are likely to be many more plant-specific examples awaiting discovery.

Fluorescence dye concentrations, autofluorescence, and background levels all contribute to final output label intensities (Dumur *et al.*, 2019). To account for all these factors, TCCF calculations were used to compare mRNA label intensities of the probe sets tested (McCloy *et al.*, 2014). This revealed that the *TIP41* probe set generated ~1.5 times higher relative label intensity than the other sets (Fig. 4C). However, it should be noted even *TIP41* smFISH RNA labelling generates much lower fluorescence compared with dyes such as DAPI or amplified conjugated secondary antibodies. For lower intensity fluorescence, wide field microscopy has often been found to provide better resolution than confocal systems (Stelzer, 1998). Although confocal microscope imaging capabilities are commonly considered superior to widefield systems, theoretical optical scenarios used for comparison are simply not achievable in practice (Sheppard and Choudhury, 1977; Brakenhoff *et al.*, 1979). This conclusion has been consistent with our experience of probe detection. However, Zhao *et al.* (2022, Preprint) have recently reported an smFISH method that can be carried out on fixed, optically cleared reporter lines. This method enables simultaneous RNA and fluorescent protein detection by confocal microscopy in whole-mount Arabidopsis roots, leaves, and embryos. The reference probe sets reported here are compatible with this new method as they broadly have invariant expression during plant development. Therefore, where maintenance of organ architecture or retention of fluorescent protein labelling is a priority, we recommend using one of our reference sets to facilitate quantitative 3D whole-mount smFISH.

Our simplified protocol and reference gene sets provide inexperienced researchers with the tools and knowledge required to set up quantitative smFISH imaging experiments. We anticipate that uptake of our method together with the recently published whole mount version (Zhao *et al.*, 2022, Preprint) and development of live RNA imaging approaches will be pivotal for filling the many knowledge gaps that currently exist regarding plant RNA metabolism and localization.

## Supplementary data

The following supplementary data are available at *JXB* online.

Fig. S1. Root apical meristem cells suitable for smFISH quantitative studies.

Fig. S2. Positive control polyA RNA labelling with T30 probes provides cell outlines.

Fig. S3. Representative control images for root apical meristem cell smFISH.

Fig. S4. Dual dye MON1 mRNA labelling using odd and even probe sets.

Fig. S5. Comparable cell sizes included in each experimental group.

Fig. S6. Variation observed for *PP2A* mRNA cellular concentrations.

Table S1. smFISH probe sequences.

Table S2. Data supporting the findings of this study.

erac521_suppl_Supplementary_Figures

erac521_suppl_Supplementary_Table_S1

erac521_suppl_Supplementary_Table_S2

## Data Availability

All data supporting the findings of this study are available within the paper and within its supplementary materials published online. Raw microscope images are available at the Dryad Digital repository https://doi.org/10.5061/dryad.vt4b8gtvp (Duncan *et al.*, 2023). The detailed protocol for Arabidopsis root meristem smFISH is available at Protocols.io (dx.doi.org/10.17504/protocols.io.rm7vzyworlx1/v1).
