# Supplementary material for: Reference genes for quantitative Arabidopsis single molecule RNA fluorescence in situ hybridization
Source: J Exp Bot. 2022 Dec 29;74(7):2405–15. doi: 10.1093/jxb/erac521 (PMC10082928; doi:10.1093/jxb/erac521)
Supplement: erac521_suppl_Supplementary_Figures [file erac521_suppl_supplementary_figures.pdf]

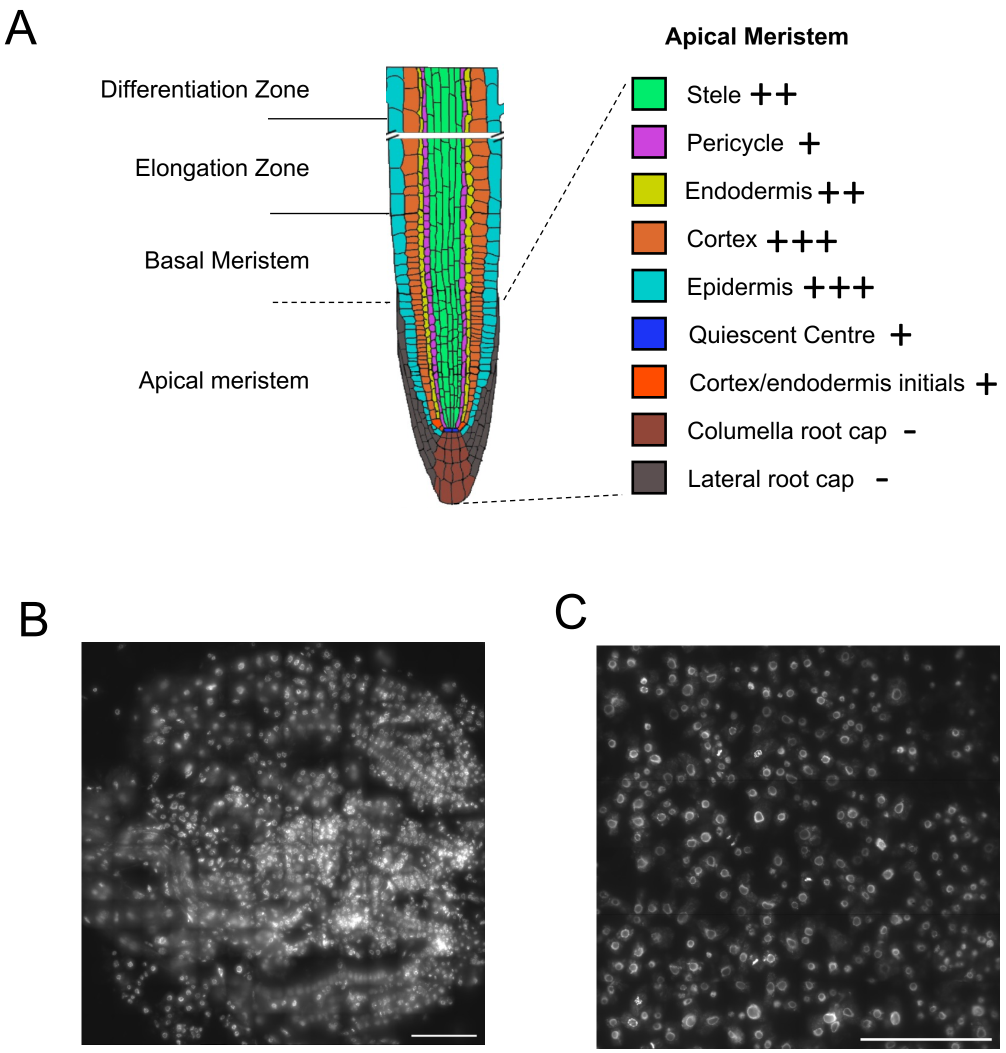

Supplementary Figure 1. Root apical meristem cells suitable for smFISH quantitative studies. A. An Arabidopsis root diagram indicating estimated proportions of cell types included as part of our smFISH study (root figure adapted from (De Smet *et al*., 2015)). **B.** An overview of a root apical meristem squash. **C.** An example of a single cell layer region best suited to quantitative smFISH analysis. Grey = DAPI stain. Scale bars in B and C = 100 μm.

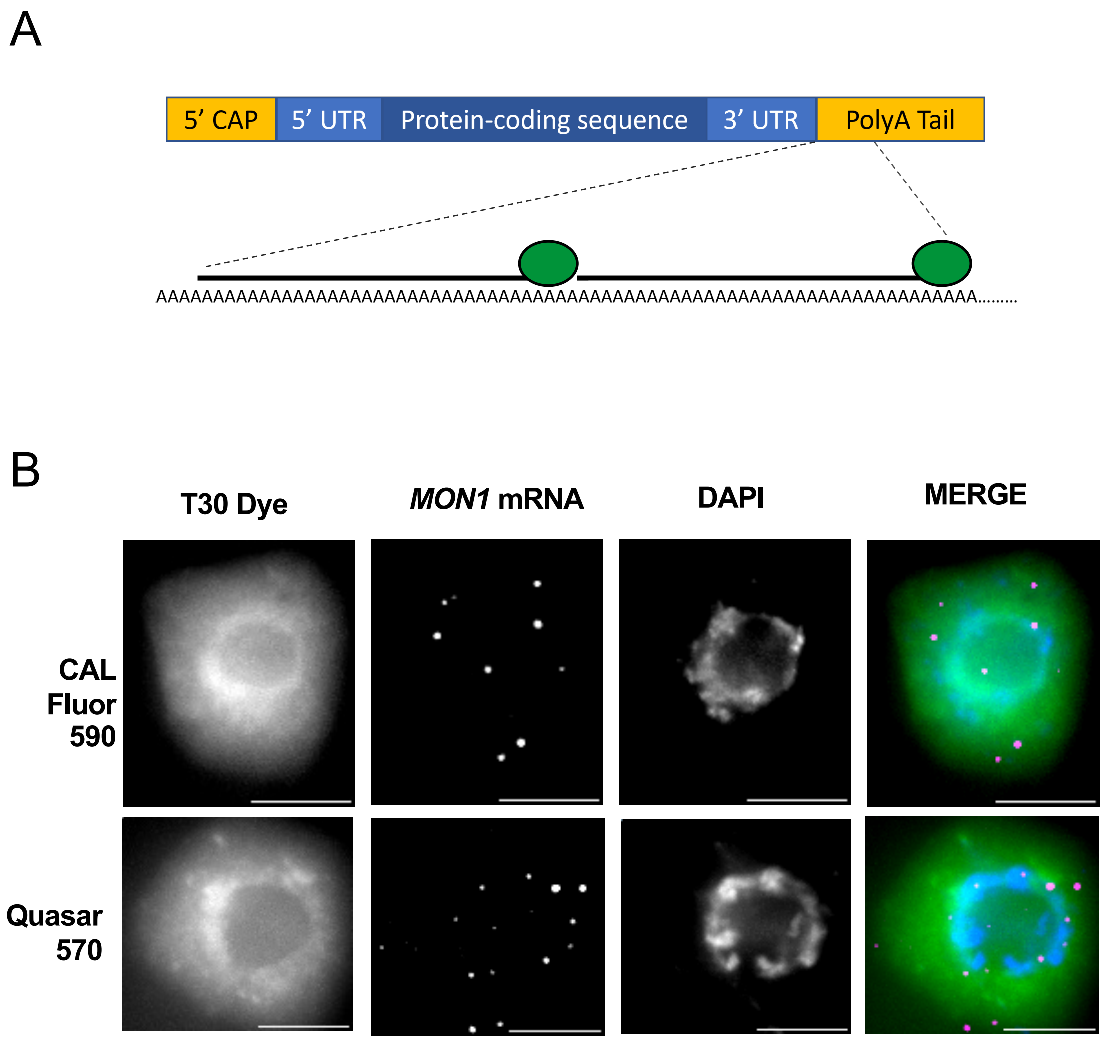

Supplementary Figure 2. Positive control polyA RNA labelling with T30 probe sets provide cell outlines.

A. Messenger RNA model highlighting T30 probe binding sites on polyA tails. **B.** Maximum z projected stacks of representative Col-0 root apical meristem cells labelled using T30 probe sets conjugated to either Quasar 570 or CAL Fluor 590 dye. Both sets were multiplexed with *MON1* mRNA probes. Pseudo colours in the merged images: Green = T30 probes, Magenta = *MON1* mRNA and Blue = nuclear stain DAPI. Rolling ball subtraction was applied to *MON1* mRNA images to improve clarity. Scale bars = 5 µm.

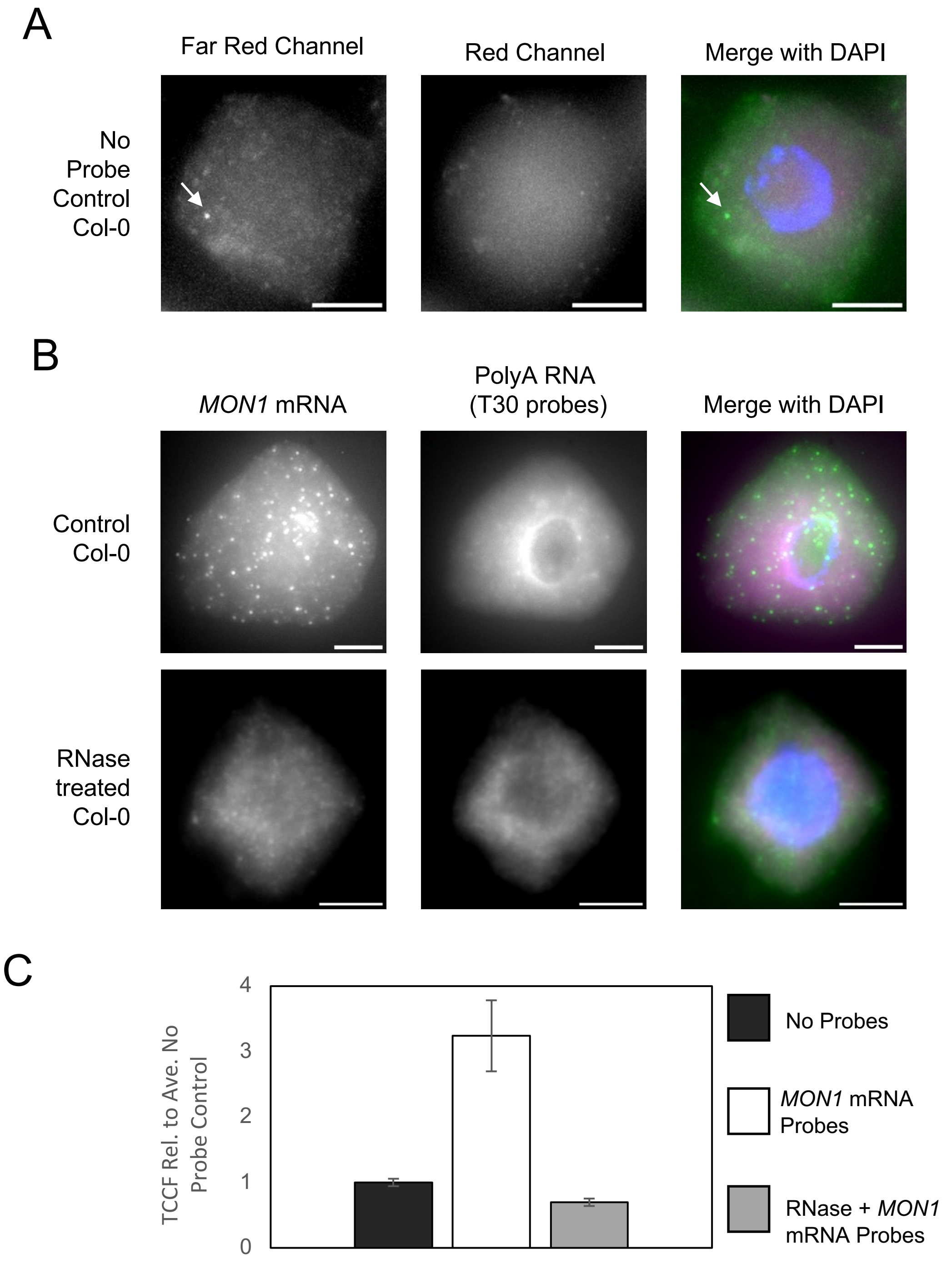

Supplementary Figure 3. Control experiments for root apical meristem cell smFISH.
A. Images acquired following the smFISH protocol without any probes. The white arrows indicate a false positive signal. **B.** *MON1* mRNA (Quasar 670 dye) and T30 polyA RNA (Quasar 570 dye) probes imaged with and without RNase treatment. **C.** Integrated density relative to no probe control shows the extent of probe fluorescence depletion following RNase treatment. Error bars represent +/- SD and *n* = 12. Pseudo colours used in the merged images: Blue = nuclear stain DAPI, Green = Far red channel in A and *MON1* mRNA probes in B (Quasar 670), Magenta = Red channel in A and T30 probes (Quasar 570) in B. Scale bars = 5 µm.

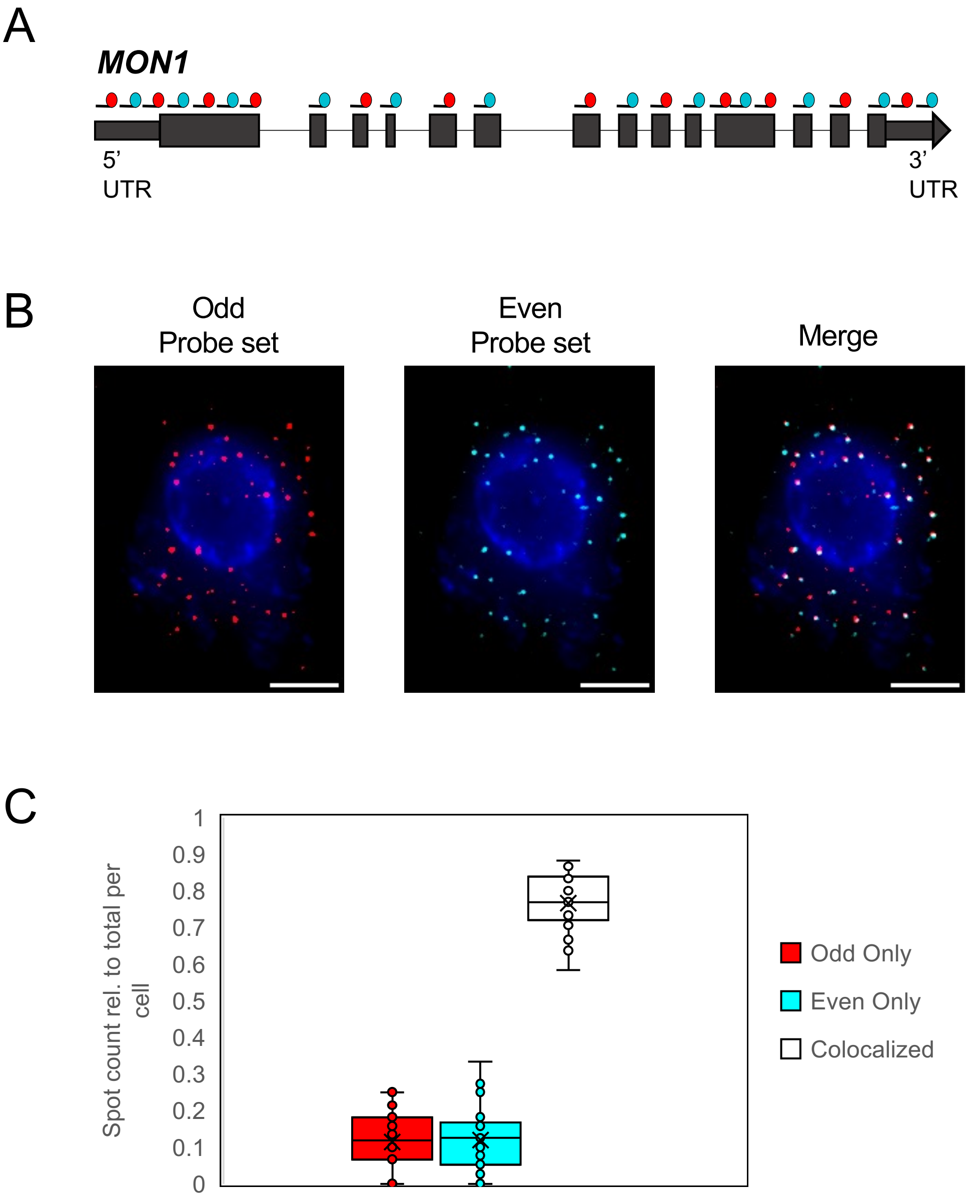

Supplementary Figure 4. Dual dye *MON1* mRNA labelling using odd and even probe sets. A. *MON1* gene model indicating alternating probe dye positions. **B.** Representative max Z projected root apical meristem cell image: Red = *MON1* mRNA odd probes (Quasar 570 dye), Cyan = *MON1* mRNA even probes (ATTO 647N dye) and Blue = nuclear stain DAPI. Rolling ball subtraction was applied to *MON1* mRNA images to improve clarity. **C.** Box plots showing the number of individual and co-localized spots detected relative to the total detected per cell. The whiskers indicate minimum and maximum values and each box indicates the interquartile range and contains a median line. Scale bar = 5 µm. n = 449 spots in total.

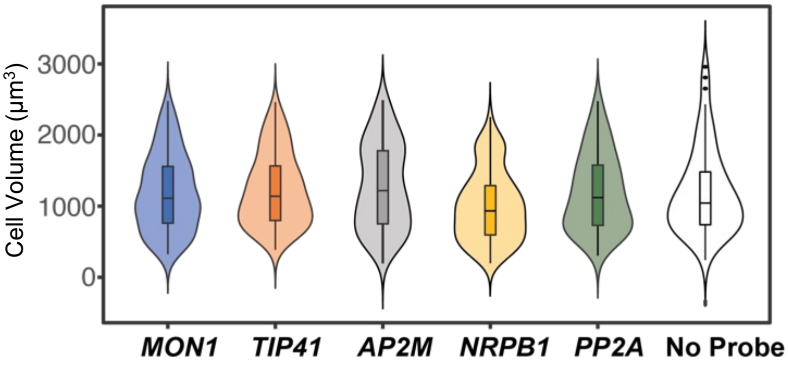

Supplementary Figure 5. Comparable cell sizes included in each experimental group. Violin plots showing the range of root apical meristem sizes included in each experimental group. Box plot sections indicate the median, 25th and 75th percentiles. Whiskers indicate minimum and maximum values.

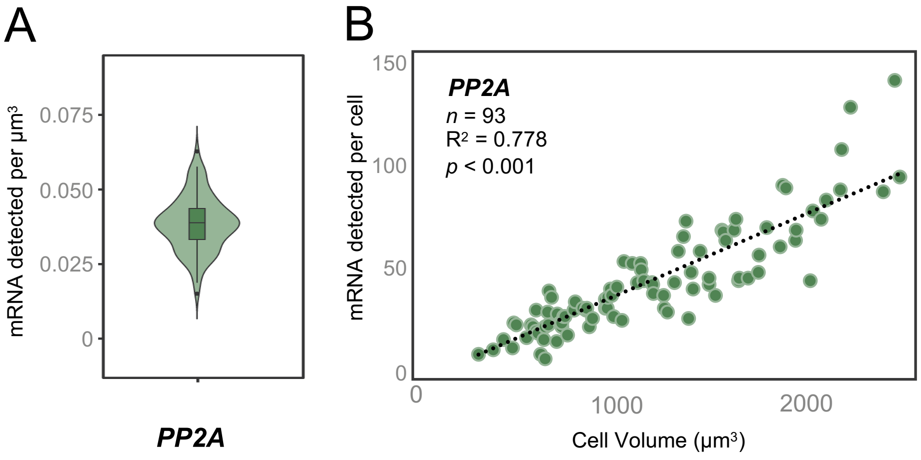

Supplementary Figure 6. Variation observed for *PP2A* mRNA cellular concentrations. A. Violin plot showing data distribution of *PP2A* mRNA detected per cellular μm^3. Box plot sections indicate the median, 25th and 75th percentiles. Whiskers indicate minimum and maximum values. **B.** Scatter plot showing variation of mRNA per cell across a range of root apical meristem cell volumes together with Pearson's correlation coefficient statistics.
